# Time lag between oscillatory pressure and flow affecting accuracy of forced oscillation technique

**DOI:** 10.1186/1475-925X-10-65

**Published:** 2011-07-29

**Authors:** Junichi Ohishi, Hajime Kurosawa

**Affiliations:** 1 Department of Occupational Health, Tohoku University Graduate School of Medicine, Sendai, 980, Japan

## Abstract

**Background:**

The forced oscillation technique (FOT) is a simple method for assessing the oscillatory mechanics of the respiratory system. The oscillatory properties, respiratory system resistance (Rrs) and reactance (Xrs), are calculated from the oscillatory pressure/flow relationship. Although the FOT has been a well-established technique, some detailed experimental conditions would be different among institutions.

**Methods:**

We evaluated whether time lags produced by the experimental conditions such as different positions of the sensors can affect the accuracy of the FOT. If the position of the pressure sensor is different from the flow sensor, a time lag may occur in the measurements. The effect of the time lag was studied by numerical analysis.

**Results:**

Rrs was estimated to be increased and Xrs decreased with an increase in the time lag, especially at a high oscillatory frequency of the medium-frequency range (5-35 Hz). At the high-frequency range (10-500 Hz), Rrs and Xrs were strikingly different in the values of the time lag.

**Conclusion:**

A time lag between the oscillatory pressure and flow may be involved in the accuracy of the FOT, suggesting that it needs to be minimized or compensated for with signal processing. Researchers should pay attention to such detailed experimental conditions of the FOT apparatus.

## 

The forced oscillation technique (FOT) is a simple method for assessing the oscillatory mechanics of the respiratory system, which has provided important findings in respiratory physiology [1,2]. Currently, the FOT using multi-frequency signals such as pseudo random noise or impulse signals is widely used in clinical research [3-6]. Although the FOT has been a well-established technique, some detailed experimental conditions would be different among institutions. To elucidate the effects of the experimental conditions may be important for more accurate FOT measurements. The purpose of this study was to evaluate whether time lags produced by the experimental conditions such as different positions of the sensors can affect the accuracy of the FOT.

The oscillatory properties, Rrs as the resistive component and Xrs as the reactive one, are calculated by a spectral relationship between the oscillatory pressure and flow. Generally, the oscillatory pressure and flow are measured simultaneously by sensors such as a pressure transducer or pneumotachograph near the subject's mouth, which are analyzed in the signal processing by Fourier transform [7]. In some previous papers, the sensors in the diagram were at different distances from the subject's mouth [2-4]. This difference would cause a time lag in the oscillatory pressure and flow in the signal processing. To evaluate whether the time lag could affect the measurement of the FOT, this effect was examined by numerical analysis as follows.

The respiratory impedance of a subject (Z_sub_) is often represented by a simple linear model of the resistance (R), inertance (L), and compliance (C). That is,(1)

where ω = 2πf (f = oscillatory frequency), . If the flow signals (f) are recorded τ seconds earlier than the pressure signals (p) due to differences in the positions of the sensors, the respiratory impedance (Zrs) is calculated as follows,(2)

The resonant and cutoff frequencies (f_0 _and f_c _respectively) are the properties of Xrs, the former of which is used to evaluate the respiratory system. F_c _is only used to determine the Xrs-curve versus oscillatory frequency, which is not actually used in the FOT. Those parameters are defined by(3)

Then, Rrs and Xrs are given by(4)(5)(6)

Here, if R = 1 (standard value without units), f_0 _= 8 Hz, f_c _= 3 Hz, and τ varies from 0 to 600 μ seconds (every 200 μ seconds), Rrs and Xrs versus the medium oscillatory frequency (from 5 to 35 Hz) are as shown in figure 1. Rrs was estimated to be increased and Xrs decreased with an increase in τ, especially at the high oscillatory frequency of this range. Figure 2 shows those properties versus a high oscillatory frequency (from 10 to 500 Hz). Rrs and Xrs were strikingly different in the τ values in this frequency range. These results indicate that the time lag between the oscillatory pressure and flow can affect the oscillatory properties, suggesting that errors in the FOT measurements would occur. If the oscillatory pressure is regarded as the transmission of air, a time lag of 200 μ seconds would correspond to a distance of approximately 6.86 cm between the two points (under the condition in which the speed of sound is 343 m/s). Such a distance between the sensors may cause a time lag of several hundred microseconds, which can affect the accuracy of the FOT.

**Figure 1:**
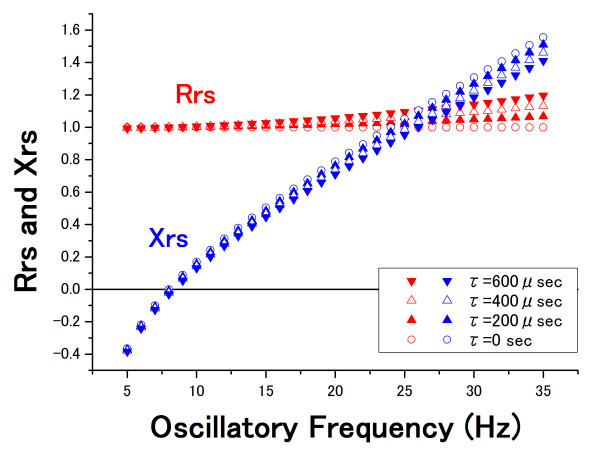
**Effect of the time lag at a medium-frequency range**. Rrs and Xrs at a frequency range from 5 to 35 Hz were calculated from equations 5 and 6, respectively. The time lag (τ) varies from 0 to 600 μ seconds (every 200 μ seconds).

**Figure 2:**
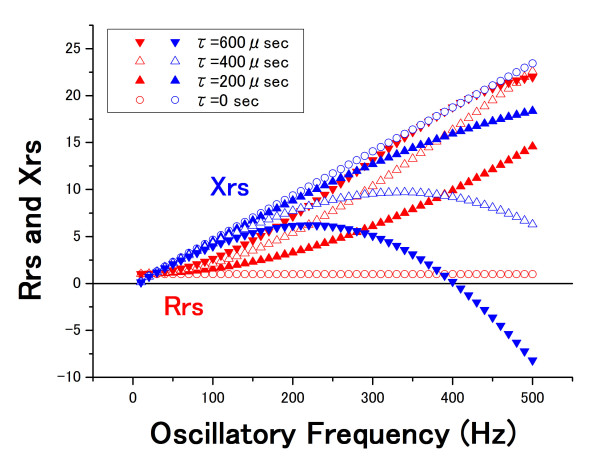
**Effect of the time lag at a high-frequency range**. Rrs and Xrs at a frequency range from 10 to 500 Hz were calculated from equations 5 and 6, respectively. The time lag (τ) varies from 0 to 600 μ seconds (every 200 μ seconds).

In conclusion, the time lag between the oscillatory pressure and flow may affect the accuracy of the FOT. This result suggests that the time lag needs to be minimized or compensated for in the signal processing for the optimal FOT measurement. Researchers should pay attention to such detailed experimental conditions of the FOT apparatus.

## Competing interests

The authors declare that they have no competing interests.

## Authors' contributions

OJ performed the analysis and drafted the manuscript. KH conceived of the study and participated in its design. All authors read and approved the final manuscript.
